# Scaling participation in payments for ecosystem services programs

**DOI:** 10.1371/journal.pone.0192211

**Published:** 2018-03-09

**Authors:** Michael G. Sorice, C. Josh Donlan, Kevin J. Boyle, Weibin Xu, Stefan Gelcich

**Affiliations:** 1 Department of Forest Resources & Environmental Conservation, Virginia Tech, Blacksburg, Virginia, United States of America; 2 Advanced Conservation Strategies, Cordoba, Spain; 3 Department of Ecology & Evolutionary Biology, Cornell University, Ithaca, New York, United States of America; 4 Department of Agricultural and Applied Economics, Virginia Tech, Blacksburg, Virginia, United States of America; 5 Center for Applied Ecology and Sustainability (CAPES), Pontificia Universidad Católica de Chile, Santiago, Chile; 6 Center for the Study of Multiple-Drivers on Marine Socio-Ecological Systems, Pontificia Universidad Católica de Chile, Santiago, Chile; 7 Bren School of Environmental Science and Management, University of California Santa Barbara, Santa Barbara, California, United States of America; Public Library of Science, UNITED KINGDOM

## Abstract

Payments for ecosystem services programs have become common tools but most have failed to achieve wide-ranging conservation outcomes. The capacity for scale and impact increases when PES programs are designed through the lens of the potential participants, yet this has received little attention in research or practice. Our work with small-scale marine fisheries integrates the social science of PES programs and provides a framework for designing programs that focus *a priori* on scaling. In addition to payments, desirable non-monetary program attributes and ecological feedbacks attract a wider range of potential participants into PES programs, including those who have more negative attitudes and lower trust. Designing programs that draw individuals into participating in PES programs is likely the most strategic path to reaching scale. Research should engage in new models of participatory research to understand these dynamics and to design programs that explicitly integrate a broad range of needs, values, and modes of implementation.

## Introduction

Payment for ecosystem services (PES) and other conservation incentive programs have become increasingly common over the past two decades, but they have struggled to attract the necessary participation and scale to the extent needed to achieve broad-level impacts. Historically, architects of these programs have focused on factors that ensure and maximize the desired programmatic and environmental outcomes, such as conditionality, additionality, and cost-effectiveness [1–4]. PES programs continue to embrace payment level as the key driver influencing participation despite evidence that payments are only one of a number of motivating factors [5]. While the relationships between program components and conservation outcomes are important [6, 7], without sufficient participation, voluntary PES programs will not scale. The necessary social science is rarely conducted *a priori* to understand the conditions under which a program will attract widespread support and participation. We offer a new way to think about participation in PES programs that employs a design perspective to enhance the capacity for a program to scale.

While the needs of a species or ecosystem service receive substantial attention during the design phase of a PES program, human needs are largely assumed to be satisfied by conditional cash payments. However, the ability to scale a conservation program is substantially greater when PES programs also create reinforcing feedbacks via social norms and ecological outcomes that stimulate social contagion effects [8, 9]. Viewed through the lens of the participant, enrollment and engagement rests on the concept of program desirability, which is a combined function of some value transfer mechanism (e.g., payment, service, or good) [10], program structure and administration [11], and information feedbacks that signal the collective efficacy of participation [12]. In addition to self-enhancing motives for external rewards (e.g., income), program desirability increases when it incorporates needs related to self-determination, understanding, trust, and feelings of belongingness [13]. Collectively, the elements of a program convey a value proposition to potential participants. A greater focus on program desirability is a critical missing factor for scaling PES programs because it ultimately frames what is desired and consequently what is achievable in a given socio-political context.

Our work with fishing communities in Chile illustrates the potential for program desirability to create a scalable PES program. Chilean small-scale fisheries provide a relevant case study: a long-standing national policy gives the government the authority to assign territorial user rights (TURFs) to fisher associations for the exclusive acess to harvest benthic resources [14]. Rights-based management approaches such as TURFs are increasingly being promoted globally to enhance the sustainability of small-scale fisheries by addressing the problem of overexploitation linked to open access fishing regimes. Voluntary programs in which fishing communities receive payments or other benefits to declare and enforce private, no-take marine reserves within a TURF (TURF reserves) are gaining traction as an approach to couple fisheries management with biodiversity conservation. With over 700 TURFs along the Chilean coast, widespread participation could result in a network of marine reserves and seascape-scale biodiversity benefits [15, 16].

**Fig 1:**
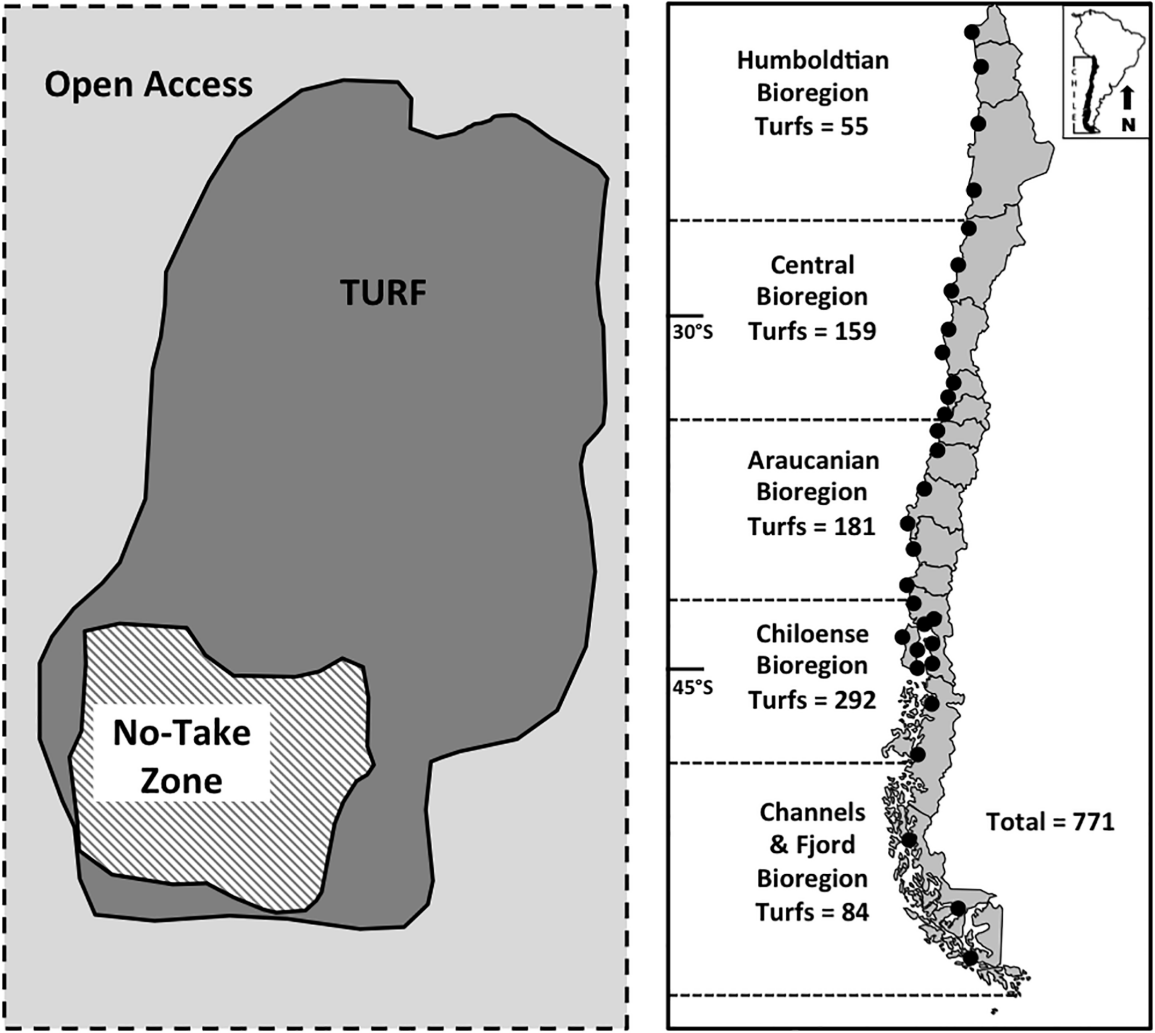
(A) A TURF-reserve program compensates Chilean fishing associations annually for setting aside a portion of their formal fishing grounds as a no-take reserve. The fishing association conducts anti-poaching surveillance. A third-party video-monitoring system monitors the no-take reserve for any contract breach. Baselines and control sites are established and biodiversity is monitored to document outcomes. (B) Some of the over 700 TURFs along the coastline of Chile showing coverage of all bioregions. Our study was conducted in the Central bioregion.

There is a growing recognition of the potential benefits of TURF-reserves including increased species richness, biomass, and densities of reef fish and macro invertebrates as well as increased environmental stewardship and monitoring [17]. A pilot program currently underway in Chile creates marine reserves within individual TURFs [18]. The pilot TURF-reserve program works by compensating small-scale fishers for the opportunity costs forgone from setting aside and enforcing a portion of their TURF as a no-take reserve (Fig 1). The program employs a human-centered design focus which places fisher needs at the forefront of the program design process while emphasizing the integration of program desirability with the ecology of benthic species.

Our research examined factors associated with program desirability that facilitate program scalability. By conducting social science research prior to undertaking efforts to scale the program, we sought to gain an understanding of the values, needs, and preferences of fishers that informs the program design in the scaling effort. We asked fishers across Chilean fisher associations to evaluate prototypes of TURF-reserve programs that differ on program characteristics related to program structure and the efficacy of achieving resource outcomes. By jointly considering payments and nonmonetary program factors, we can better understand how fishers perceive the overall value of participating in a program. We expect programs aligned with the preferences and needs of fishers will garner greater approval than those that are not. Further, we expect the greater participation occurs because desirable programs draw in fishers who have more negative attitudes and trust in the program.

## Methods

### Sampling

The sample frame included members of all 12 fishing associations with functioning TURFs in administrative Region V in the Central bioregion of Chile (Fig 1). We conducted face-to-face structured surveys with members of fisher associations at each site through direct encounter convenience sampling strategy. We interviewed a maximum of 14 fishers per association, which represented 12% to 70% of association members. The average sample of members was 25% with an average number of surveys per fishing association of 11. The president of the association was always interviewed. The elicited information included preferences for a proposed TURF-reserve program, attitudes toward participating, institutional trust, and information about the role of fishing as part of their livelihood. This study was approved by the Pontificia Universidad Catolica and Virginia Tech institutional review boards.

### Measuring program desirability

The TURF-reserve program was described to respondents as a marine biodiversity conservation program in which businesses, organizations, and agencies interested protecting marine biodiversity, either for philanthropic purposes or to offset environmental impacts elsewhere, would provide the fishing association with an annual cash payment to set aside 15 hectares of their fishing territory as a no-take protected area. We selected 15 hectares based on long-term ecological research demonstrating that a 15 hectare no-take marine protected area (MPA) in similar habitat can sustain a functioning marine ecosystem [19]. Further, the only well-enforced MPA in Chile (Las Cruces) with long-term data on recovery is 15 hectares. Fishers were informed that an independent nonprofit organization would be created for the sole purpose of administering the program. Although the fishing association would actually receive the cash payment, each member would receive a portion of the payment.

#### Program attributes

Because the actual behavior of participating in a TURF-reserve program cannot be observed *a priori*, we asked fishers to indicate their approval of program prototypes, which involved the designation of a no-take zone within a fishing association’s TURF [18]. The program prototypes consisted of 3 program structure attributes (i.e., contract length, annual payment, and monitoring requirements) and 2 expected efficacy attributes (i.e., increase in an economically important species and increase in variety reef fish species) (Table 1).

**Table 1:** TURF-reserve program characteristics and levels evaluated. Levels in bold were empirically identified as the most desirable program conditions from the fishers’ perspectives.

Program Characteristics	Levels	
Contract length (period of no take)	**2 years**	
	6 years	
	10 years	
Annual payment to TURF for participation	USD	CLP
	$2,750	1,800,000
	$4,130	2,700,000
	$5,500	3,600,000
	$6,730	4,400,000
	**$8,100**	**5,300,000**
Anti-poaching monitoring requirements	Coastal video	
	Coastal video & boat-based surveillance	
Increase in targeted resource (loco)	No change (0%)	
	Moderate increase (10%)	
	**High increase (20%)**	
Increase in biodiversity (reef fish)	No change (0%)	
	**Moderate increase (10%)**	
	High increase (20%)	

Longer contracts increase the likelihood of environmental benefits and the opportunity costs of reduced access and fisheries revenue. The primary economic species of interest, loco (*Concholepas concholepas*) is a benthic marine invertebrate and the commercial species historically most targeted by small-scale fishers within TURFs. It can recover within 2–3 years from overharvesting once humans are excluded [20]. We included a 10-year contract to account for the biodiversity conservation benefits of a longer-term contract, as well as to capture some of more slowly-unfolding ecological dynamics—including potential spillover effects from significant changes in loco production [21].

Monetary payments provide an external inducement to motivate conservation behavior by compensating fishers for the opportunity cost of foregone harvesting opportunities. The range of annual payments was based on past focus groups, consultation with fisher association leaders, and the values associated with enforcing TURFs. We used an average of 3.6 million Chilean pesos as a reasonable yearly enforcement cost for a TURF, which is equivalent to having two guards per month on a minimal monthly wage in Chile. To create a range of payments that would be perceived as high and low, we created additional payments that differed by ±25% and ±50% from the 3.6 million pesos. We converted Chilean pesos to US dollars using the average exchange rate for 2015 of 645:1.

A centerpiece of a TURF-reserve program is a contractual relationship with participating fishing associations that dictates explicit behaviors: exclusion of harvesting activity by TURF members and regular anti-poaching surveillance to prevent harvesting from non-members. Because TURFs (and the designated no-take zones) are located in near-shore coastal areas, land-based video-monitoring is a viable option [see 18]. Thus, we provided two scenarios for monitoring requirements: regular boat-based monitoring accompanied by stationary coastal video monitoring and the latter alone.

Finally, because information feedbacks about collective efficacy of environmental protection programs are a key element in achieving collective action [22], we examined how a TURF-reserve program’s resource outcomes influence participation via two perspectives: fishers’ self-enhancement motivation (i.e., a commercial species) and prosocial motivation (i.e., reef fish as an indicator of biodiversity benefit). These respectively reflect use and nonuse values for fisheries resources [23]. Loco represents an economically valuable species and fishers do not commonly harvest reef fish within TURFs. In both cases, we expected the program to maintain or increase both factors.

We employed a choice experiment approach to assess fisher preferences for programs [24]. With this approach, it is both impractical and statistically inefficient to include all possible combinations of attributes in Table 1 (in our case, 216). We selected a subset of 60 combinations using a main-effects design in which each of the attributes is orthogonal to the other (a fractional factorial design) [24]. In the survey, each fisher responded to four sets of these scenarios blocked into 15 versions of the survey.

#### Fisher characteristics

We also considered the influence of three characteristics of small-scale fishers on participation: 1) attitudes toward a TURF-reserve program, including beliefs about outcomes of participating; 2) trust that the facilitating conditions exist for the program to be successfully implemented; and 3) livelihood dependence on fishing. Attitudes toward participating are a key predictor of behavior [25] and have been shown to be important for understanding the success of TURF management [26]. Trust in key institutions to support new initiatives is a critical component in the success of co-management governance approaches [27]. Livelihood dependence reflects the degree to which fishers are economically and socially reliant on fishing. This includes income as well as the sense of identity and self-esteem derived from engaging in fishing, and the capacity to cope with change [28].

Employing attitude theory, we asked fishers to evaluate the merits of the TURF-reserve program based on two instrumental indicators (*Foolish* to *Wise* and *Ineffective* to *Effective*) and two evaluative indicators (*Undesirable* to *Desirable* and *Bad* to *Good*) [25]. We also asked fishers to provide an evaluation of the risk associated with participating (*Risky* to *Safe*). Because fishers may hold differing beliefs about how participating in the program might affect their income, we included their belief about the expected change in their income as a result of participating. Participants were asked if enrolling in the program would decrease, increase, or have no effect on their income.

Our indicators of trust focused on the enabling conditions present in the current social and political setting. Fishers indicated how much they trusted different groups who may be involved the TURF-reserve program (*Distrust entirely* to *Trust entirely*). The target groups included fishing association leaders, consultants that often conduct assessments with fishing associations, police or security that might enforce poaching events, local government, fisheries officers, and fisheries scientists.

Having a shared (i.e., collective) identity with one’s organization creates an enhanced willingness for members to cooperate in conservation actions [29], and trust the association to make decisions about fisheries management [30]. We employed two commonly used indicators of collective identity (*I feel that the [association’s] successes are my successes* and *When I talk about the [association] I usually say ‘we’ instead of ‘they’* [31].

Trust in the association to engage in TURF-reserve program is also a function of an individual fisher’s involvement in the association, as well as the individual’s trust in the association’s leadership. We asked fishers to indicate their involvement in association issues in general, as well as in natural resource decisions (*Almost no involvement* to *High involvement*). They also provided an overall evaluation of their trust in the fishing association’s leaders.

Reliance on a narrow range of natural resources or single ecosystem can lead to social and economic stresses within one’s livelihood due to a limited ability to adapt to environmental changes triggered by volatility or shocks. It is a key antecedent to the social resilience of social-ecological systems [32], and can be operationalized as perceptions of economic and social dependency on one’s livelihood [33]. We employed seven indicators to assess key antecedents to resource dependency: centrality of fishing to one’s lifestyle, self-identity, economic independence, and perceived attachment to the fishing livelihood (see S1 Table).

### Data analysis

We modeled program participation as a function of the TURF-reserve program and fisher characteristics. We focused our attention on the conditions that would yield a simple majority decision to approve participation (i.e., >50% approval) because this is the adoption rule used by fisher associations.

Our model assumes that fishers are willing to approve a TURF-reserve program if the utility of participating is greater than not participating. Further, fishers will approve a program in which their overall preference for program structure and expected outcomes exceeds an alternative program. That is fishers prefer program *i* over *j* when *U*_*i*_ > *U*_*j*_. The utility function is unobservable and instead we model the probability of choosing program *i* over *j* using a conditional logit formulation [34]:

$$P(i|i\in M)=\frac{e^{V_{i}}}{\sum_{j=M}e^{V_{j}}}$$ (1)

where *M* is all of the choice sets contained in the study, *V*_*i*_ and *V*_*j*_ are the observable utilities for each set of programs. Marginal effects were calculated at representative values of covariates using *Stata* (version 13), and we report them as predicted probabilities [35].

To reduce the number of redundant parameters in the final model, we used nonmultidimensional scaling followed by similarity profile analysis, a non-parametric statistical test to identify groupings for each set of attitudes, trust, and livelihood variables [36]. We conducted an all-possible subsets conditional logit and selected the most parsimonious model with the lowest AIC.

**Fig 2:**
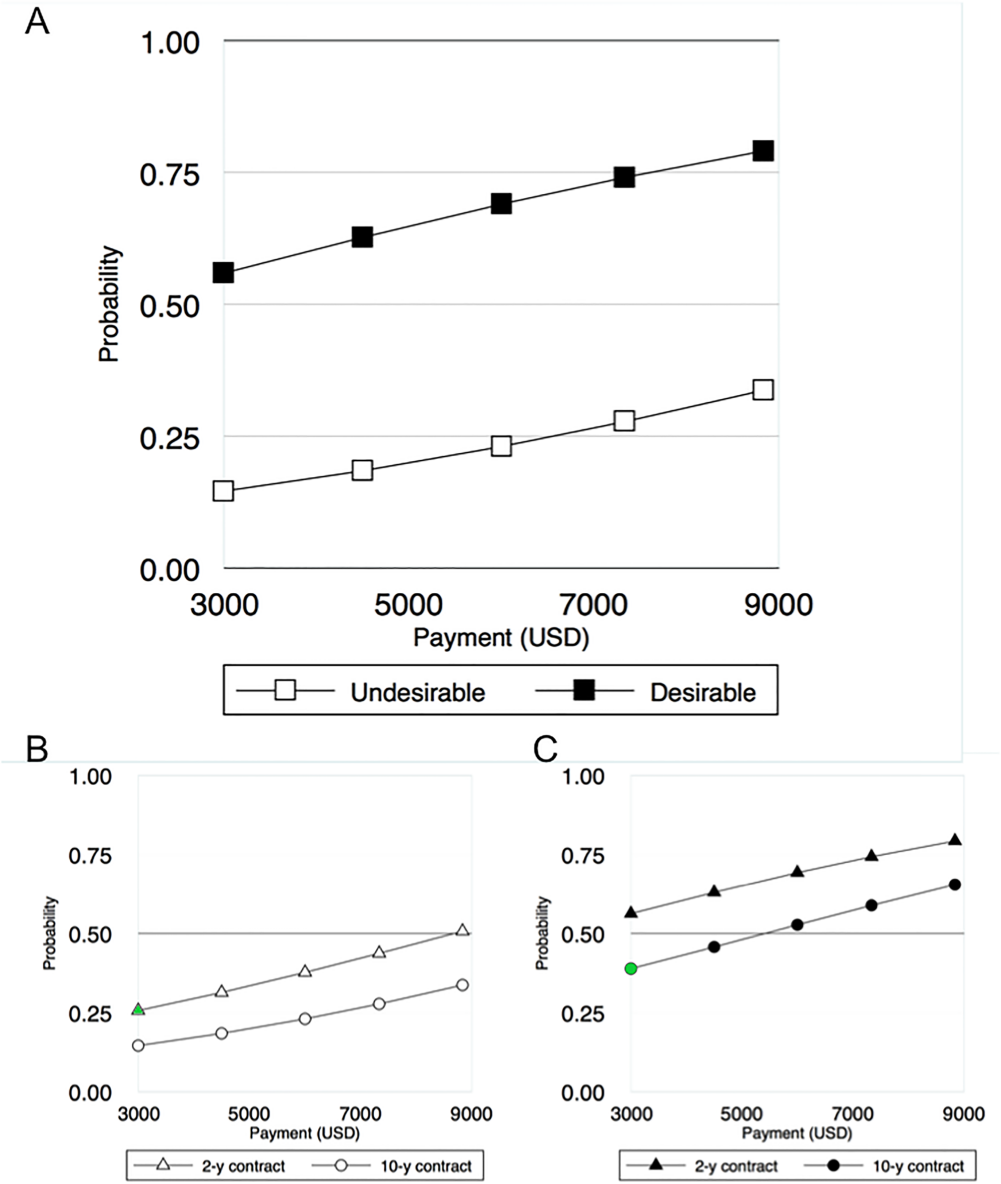
(A) Predicted probability of approving a TURF-reserve program between the most and least desirable program, based on fisher preferences or program characteristics and outcomes. Payments have a positive effect on approval for both programs, but approval differs drastically, with the undesirable program never reaching majority (50%) approval. The effect of contract length on approval for undesirable (B) and desirable (C) programs reveals a tradeoff (highlighted in green): the probability of accepting an unfavorable 10-year contract at US$2,750 per year (probability = 0.39) in an otherwise desirable program is higher than the probability of accepting a favorable 2-year contract at the same payment level (probability = 0.26) in an otherwise undesirable program.

We modeled the probability that a fisher association would approve desirable and undesirable programs across payment levels. Predicted probabilities were calculated to estimate approval for the most undesirable program and the most desirable program (Fig 2). The desirable program was created holding all choice model coefficients at their empirically-identified most preferred level. The undesirable program was created holding all choice model coefficients as their least preferred level. Covariates were held at their medians. Program adoption was assumed when approval >50% as this simple majority rule is the same rule used by fisher associations.

To examine the role of fisher characteristics and beliefs, we calculated predicted probabilities across payment levels for both desirable and undesirable programs while holding participant characteristics at representative values of low, medium, and high levels. Because these variables were measured on a continuous scale from 0 to 7, we selected the following: low = 1, medium = 3.5, high = 7. We rescaled the years fishing variable to fit a 0 to 7 scale. Combining these 3 levels each for attitude, trust, and livelihood dependence generates 27 (3^3^) possible scenarios. This overall evaluation is based on the calculation of the probability of choice for one alternative over any other alternative(s), as:

$$Pr(y_{ij})=\frac{e^{x_{ij}\beta }}{1+e^{x_{ij}\beta }}$$ (2)

The accuracy of stated preference models, of which choice questions are a primary variant, is the center of a lively debate [37, 38]. Rather than engage directly in this debate, we take the position that the relationships related to desirable and undesirable programs are the most relevant result of our study. List and Gallet [37] reinforce our finding of the importance of desirable program characteristics in achieving majority approval. Applying insights from their study to predictions in our study, the undesirable program becomes even less feasible while the desirable program can still achieve majority approval in fisher associations. Thus, robust support remains for program desirability concept.

## Results

Of the 168 fishers approached, 138 completed the entire interview for an overall cooperation rate of 82%. Similarity profile analysis reduced the number of covariates from 21 to 15. Three attitude measures were combined into a single indicator (desirable/undesirable, wise/foolish, good/bad). In the trust group, trust in police/security and fisheries officers were combined into a single indicator. The two collective identity indicators were combined as were the two measures of involvement. In the livelihoods group only two variables, Identity1 and Identity2 (see S1 Table) were combined into a single indicator. We combined variables by averaging items together.

### Program characteristics

All program characteristics, except monitoring requirements, have a significant impact on fishers’ approval of a TURF-reserve program (see S1 File). An increase in payment increased the odds of participation: for about every US$1,400 increase, the odds of approval increased by 37%. Fisher association members preferred shorter-term contracts, which is consistent with other research on conservation contracts [11, 39]. Compared to a 10-year contract, offering fishers a 6-year contract increases the odds of approval by 46%, while a 2-year contract increases the odds by 52%.

Expectations that the program will increase economically valuable loco and ecologically important reef fish have substantial influences on approval. If the TURF-reserve program increases the loco population by a moderate (10%) or large (20%) amount over the status quo, the odds of approval increases by 100% and 138%, respectively. A moderate increase in reef fish by 10% increases the odds of program approval by 50%. However, a large (20%) increase in reef fish over the status quo is not associated with an increase in program approval. This preference for only a moderate increase in reef fish may be in part because the primary reef fish species prey on benthic resources, leading to possible concerns about decreases in loco availability at high reef fish diversity [19].

No differences in predicted participation exist between stationary coastal video monitoring with boat-based monitoring versus only coastal video monitoring. We suspect that this lack of a significant effect may be due to the fact that Chilean fishing associations commonly conduct boat-based anti-poaching monitoring within the TURFs [40].

The relationship between participation and program desirability reflected by a combination of program factors and collective efficacy provides greater insight into designing PES programs to scale. We compared predicted approval for the most and least desirable programs. The most desirable program includes a 2-year renewable contract length, coastal video & boat-based surveillance, a large (20%) increase in the targeted resource, and a moderate (10%) increase in biodiversity. The least desirable program includes a 10-year renewable contract, coastal video surveillance, and no increase in the targeted resource and biodiversity.

Programs with reduced commitments and that provide greater confidence in benefits have a greater potential to scale. A TURF-reserve program with the least desirable characteristics results in a maximum approval rating of 34% at the highest payment level (Fig 2a) while the most desirable program results in approval ratings across all payment levels ranging from 56–80%. Majority approval, and thus scalability, is impossible with the least desirable program—even when the program is willing to provide large payments. In contrast, majority approval is reached with the most desirable program at the lowest payment level (Fig 2a).

When species or habitat needs do not align with program participants’ needs, tradeoffs can be identified that retain the ability to assess the potential to scale PES programs. For instance, Chilean fishers are more likely to approve a desirable TURF-reserve program with a less-preferred 10-year contract than they are to approve an undesirable TURF-reserve program that contains a more preferred 2-year contract (Fig 2b and 2c). Program desirability can enhance a program’s capacity to scale by identifying and addressing potential tradeoffs, and thus offsetting concerns of individual participants.

### Participant characteristics

**Fig 3:**
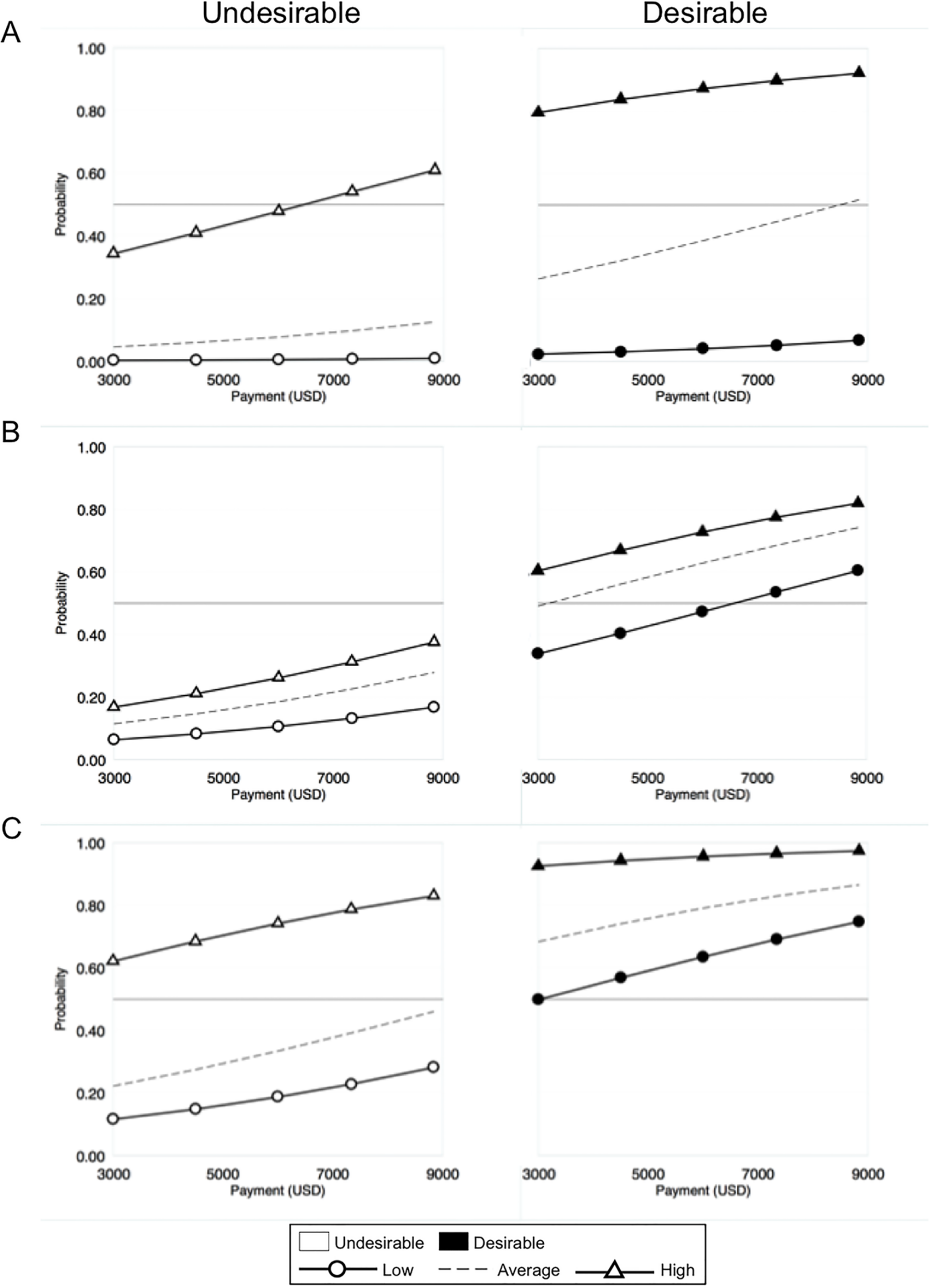
Participation probabilities based on (A) attitudes toward participation, (B) trust that facilitating conditions implement the program exists, and (C) fisher’s dependence on the resource for the livelihood.

The success of scaling a PES program is also a function of the characteristics of potential participants. Individual attitudes toward the program affect participation, as does trust in the institutions involved and the degree to which potential participants are reliant on the natural resources involved (Fig 3).

Payment levels offered to fishers have little influence in achieving majority approval if attitudes toward an undesirable program are negative. In fact, majority approval can only be achieved with the highest payment level when fishers have a positive attitude (Fig 3a, undesirable panel). In contrast, approval of a desirable program is over 75% at low payment levels for fishers with average attitudes (Fig 3a, desirable panel). Program desirability has the strongest effect on fishers with average attitudes, reaching majority approval at the highest payment level, but little effect on fishers with the most negative attitudes. These findings suggest that failure to consider the non-monetary aspects of program desirability may limit participation to the small percentage of target stakeholders that already strongly believe in the program.

The relative magnitude of payments as a factor was most apparent when attitudes were negative, trust was low, and dependence on fishing was low. For the most undesirable program, the relative approval of fishers with very negative attitudes increased by a multiplicative factor of 2.96 from the lowest to the highest payment level, but the absolute change in the probability of approval remains essentially zero (increasing only +0.6% from 0.3% to 0.9%). In contrast, the relative change in approval of fishers with very positive attitudes was a factor of 1.77 with an absolute increase in probability from 34% to 61% (+27%). Similar relationships occur for trust. These results suggest that payments are important determinants of program approval, but by themselves are not sufficient to achieve threshold levels of participation necessary to drive landscape-scale marine conservation.

Program desirability serves as a strong pull factor for a TURF-reserve program with respect to fishers’ trust that the enabling conditions exist for a program. For fishers with the highest degree of trust, majority approval is unachievable in an undesirable program, even at the highest payment level (Fig 3b). In contrast, majority approval is reached in a desirable program at the lowest payment level for fishers with average trust levels. High-trusting fishers in an undesirable program approve a high-cost program (US$8,100 per annum) at the same rate of low-trusting fishers in a low-cost desirable program (US$2,750 per annum). These results suggest strong roles for program structure and information feedbacks in achieving scale even when the trust of the target population may be lacking.

High livelihood dependence on fishing has the greatest impact on approval of a TURF-reserve program with respect to program desirability. High dependence results in majority approval at all payment levels for both undesirable and desirable programs (Fig 3c). For fishers with an average or low levels of dependence, approval favors the desirable program. For fishers with an average level of dependence on fishing, the probability of approval increased between 40–47% (Mean = 44%) across payment levels between the least and the most desirable program (Fig 3c). Similar increases are found for low-dependence fishers (Mean = 44%, Range = 38–47%). Given that implementing a no-take zone involves a high amount of uncertainty with respect to outcomes, those with the most invested in the resource are more likely to engage in a program to establish a no-take zone. This group may feel that risks associated with the TURF-reserve program could be reversed by their own work and knowledge of the system. Fishers who are more dependent on fishing in Chile have been shown to be risk-acceptant towards investing in novel marine management solutions [41]. An alternative hypothesis is that those fishers that are heavily invested in fishing are likely to be more vulnerable to variability in income, compared to other fishers who have multiple revenue streams. Thus, they may be more willing to approve a program to decrease income volatility. Nonetheless, program desirability increases the likelihood that fishers with diversified livelihoods would approve the program.

### Thresholds of approval

**Fig 4:**
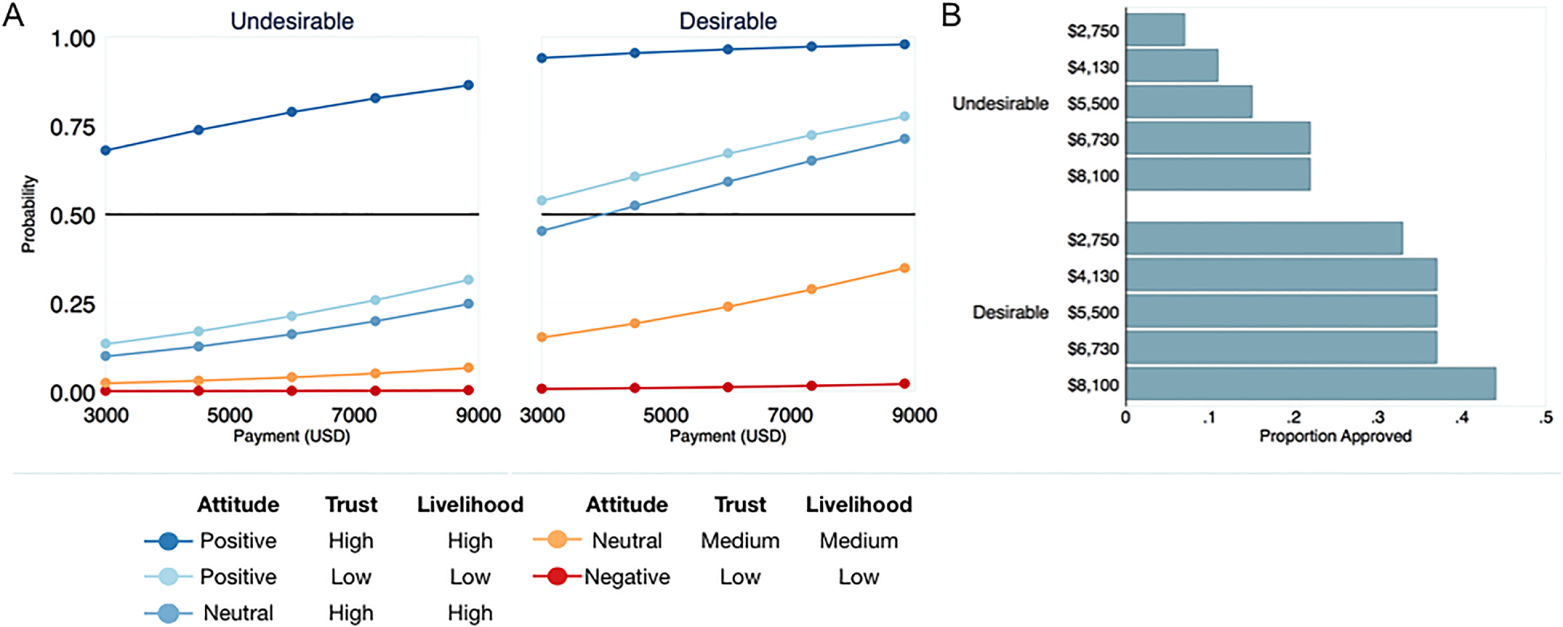
(A) PES program scenarios through the lens of potential participant characteristics. Although program desirability is not a panacea, it increases the diversity of participants. In Chile, majority program approval by fishers with low levels of trust and livelihood dependence is only possible with desirable programs (blue lines). (B) The proportion of different PES programs being approved by potential participants across different payment levels. Program approval is consistently higher with desirable programs See Supporting Information for comparison of all program scenarios.

Desirable programs decrease the threshold of participation by overcoming barriers of entry related to attitudes and levels of trust. Although fishers with positive beliefs and high trust—the so-called *low-hanging fruit*—favor the undesirable program, others tend to only approve desirable programs. For instance, the number of approved programs increases by 15% under desirable conditions because such programs are more likely to appeal to fishers with more negative attitudes, lower levels of trust, and to those who rely less on fishing for their livelihood (Fig 4 and S2 File). As important, when participants are willing to accept lower payments in exchange for a program that better aligns with their preferences, the operational costs of PES programs will be lower. In sum, program desirability enables more participation at a lower cost.

## Discussion

PES and related programs will struggle to scale unless architects move beyond a focus on payments as the most important driver of participation [42, 43]. While payments serve as a relatively strong factor to obtain fishers’ approval, their ability to gain the approval of fishers substantively diminishes as attitudes become negative, trust decreases, and dependence on fishing decreases. Compared to undesirable programs, programs that are designed to better fit fisher needs and provide confidence about expected outcomes receive greater approval from fishers who have more negative attitudes, lack trust in institutions, and have diversified income streams. The effect of expected outcomes on participation highlights the need to adequately address the lack of natural science behind the design, monitoring, and evaluation of biodiversity incentive programs [6]. Programs with strong information feedbacks between outcomes and participants will result in pulling in more participants, partly because of increased certainty and enhanced trust between fishers and the program administrators [22, 44].

Many programs have been shown to reduce environmental impacts or enhance benefits, but their long-term efficacy depends on sustained voluntary participation. An *a priori* understanding of the values, needs, and preferences of the participant enables program architects to understand how factors across multiple scales can influence participation [43], and how payment effects can be undermined by other undesirable program factors [5, 45]. Our case study suggests that payments alone are insufficient to attract enough participation by Chilean fishers to scale the program and deliver significant environmental benefits. Similarly, payments alone are unlikely to sustain renewed participation in PES programs over time [42, 46].

The scalability of PES programs rests on their feasibility and ecological viability. In this regard, designing science-driven programs with an eye for conditionality, additionality, and cost effectiveness is critical [6]. However, incorporating program desirability to draw individuals into participation is likely the most strategic path to reaching scale. Simply attracting greater participation can trigger social contagion effects within a group who perceive others as willing to cooperate, especially if early adopters are community leaders or influential connectors [47, 48]. Successful program design is a key predictor of the success of conservation programs [49], and an understanding of program participation through the lens of the participant is currently a critical missing component in PES program design. In order to design for scale, PES researchers and architects should engage in new models of participatory research to design programs that explicitly integrate a broad range of needs and values, as well as modes of implementation, that appeal to more than just an individual’s pocketbook.

## Supporting information

S1 Table Measures of livelihood dependence. (DOCX)

S1 File Model results. (DOCX)

S2 File Thresholds of Participation. (DOCX)
