# Supplementary material for: Scaling participation in payments for ecosystem services programs
Source: PLoS One. 2018 Mar 9;13(3):e0192211. doi: 10.1371/journal.pone.0192211 (PMC5844514; doi:10.1371/journal.pone.0192211)
Supplement: S1 Table — (DOCX) [file pone.0192211.s001.docx]

Table S1. Indicators of Livelihood Dependency were measured on a 0 to 7 scale from *Strongly Disagree* to *Strongly Agree*.

| Concept | Indicator |
| --- | --- |
| Centrality to livelihood | Years individual has been fishing as a profession |
| Self-Identity1 | A lot of my life is organized around fishing^a^ |
| Self-Identity2 | Fishing is an important part of who I am^a^ |
| Economic Dependence | Percent of household income from artisanal fishing |
| Occupational Dependence1 | I cannot imagine doing any job other than fishing^a^ |
| Occupational Dependence2 | I have many options if I decide to no longer be a fisher^ab^ |
| Occupational Dependence3 | I would be nervous to try a profession other than fishing^a^ |

^a^Scale: Continuous scale from 0 = *Strongly Disagree* to 7 = *Strongly Agree*

^b^Reverse coded in analysis
