# Supplementary material for: Scaling participation in payments for ecosystem services programs
Source: PLoS One. 2018 Mar 9;13(3):e0192211. doi: 10.1371/journal.pone.0192211 (PMC5844514; doi:10.1371/journal.pone.0192211)
Supplement: S1 File — (DOCX) [file pone.0192211.s002.docx]

**File S1: Model results**

We employed a best subsets modeling approach using Akaike's Information Criterion (AIC) to select the final model ([Bozdogan 1987](#_ENREF_8)). The best fit conditional logit model includes the choice model attributes and the following individual-level variables:

- Attitudes
- Combined indicators: 1) Desirability, 2)Wise/Foolish, 3) Bad/Good
- Income change (Positive/Negative)
- Trust
- Trust in association leaders
- Combined indicators: 1) Trust in fisheries officers and 2) Trust in police/security
- Combined indicators of 2 collective identity indicators
- Trust local government
- Resource dependency
- Years fishing
- Occupational Dependence 1
- Occupational Dependence 2

**Table S2.** Final conditional logit estimates. All program characteristics, except monitoring requirements, have a significant impact on fishers' approval of the proposed TURF-reserve program.

| Variable | Coefficient | SE | z | P > \|z\| | 95% Confidence Interval | |
| --- | --- | --- | --- | --- | --- | --- |
| ASC | -8.090144 | 1.292856 | -6.26 | 0.0000 | -10.624095 | -5.556192 |
| Payment | 0.002037 | 0.000326 | 6.25 | 0.0000 | 0.001398 | 0.002675 |
| Monitoring |  |  |  |  |  |  |
| Shore^a^ |  |  |  |  |  |  |
| Shore & Boat | 0.015549 | 0.116681 | 0.13 | 0.8940 | -0.213142 | 0.244240 |
| Contract Length |  |  |  |  |  |  |
| 2 years^a^ |  |  |  |  |  |  |
| 6 years | -0.081223 | 0.153677 | -0.53 | 0.5971 | -0.382424 | 0.219978 |
| 10 years | -0.704307 | 0.166150 | -4.24 | 0.0000 | -1.029955 | -0.378659 |
| Reef Fish Diversity |  |  |  |  |  |  |
| No increase (0%)^a^ |  |  |  |  |  |  |
| 10% increase | 0.436339 | 0.246329 | 1.77 | 0.0765 | -0.046457 | 0.919135 |
| 20% increase | 0.241529 | 0.144213 | 1.67 | 0.0940 | -0.041124 | 0.524181 |
| Loco Abundance |  |  |  |  |  |  |
| No increase (0%)^a^ |  |  |  |  |  |  |
| 10% increase | 0.846792 | 0.256265 | 3.30 | 0.0010 | 0.344522 | 1.349062 |
| 20% increase | 0.864898 | 0.144407 | 5.99 | 0.0000 | 0.581865 | 1.147931 |
| Participant Characteristics^b^ |  |  |  |  |  |  |
| Attitude | 0.540546 | 0.151771 | 3.56 | 0.0004 | 0.243081 | 0.838012 |
| Income Change | 0.306005 | 0.111277 | 2.75 | 0.0060 | 0.087907 | 0.524104 |
| Trust – Officers & Security | -0.036619 | 0.090864 | -0.40 | 0.6869 | -0.214710 | 0.141471 |
| Collective Identity | 0.275535 | 0.113081 | 2.44 | 0.0148 | 0.053901 | 0.497169 |
| Trust – Association | -0.060885 | 0.081411 | -0.75 | 0.4545 | -0.220447 | 0.098678 |
| Trust – Local Government | 0.003787 | 0.063482 | 0.06 | 0.9524 | -0.120636 | 0.128210 |
| Years Fishing | 0.023539 | 0.013593 | 1.73 | 0.0833 | -0.003103 | 0.050180 |
| Occupational Dependence 1 | -0.019635 | 0.063352 | -0.31 | 0.7566 | -0.143803 | 0.104532 |
| Occupational Dependence 2 | 0.251529 | 0.067265 | 3.74 | 0.0002 | 0.119692 | 0.383365 |

^a^Reference category

^b^Variable Definitions: attitude, average of undesirable/desirable, wise/foolish, bad/good attitude items; income change, perception of change in income resulting from participation, trust officers & security, average of trust fisheries officers and enforcement officers; collective identity, average of *collective identity 1* and *collective identity 2*; trust association, trust organization leaders; years fishing – years person has been fishing; occupational dependence 1; occupational dependence 2 (reverse coded).
