# Supplementary material for: Scaling participation in payments for ecosystem services programs
Source: PLoS One. 2018 Mar 9;13(3):e0192211. doi: 10.1371/journal.pone.0192211 (PMC5844514; doi:10.1371/journal.pone.0192211)
Supplement: S2 File — (DOCX) [file pone.0192211.s003.docx]

**File S2: Thresholds of Participation**

Employing the TURF associations' simple majority decision rule, 5 of the 27 programs examined (18%) would be approved under the undesirable program scenario (Fig S1). Only 2 programs would be approved at the lowest cost to the program provider (US$2,750 per annum) (Fig S1 left panel). In contrast, 9 programs (33%) would be approved under the most desirable program and 75% of those would be approved at the lowest cost (Fig S1 right panel.). A greater proportion of desirable programs would consistently be approved compared undesirable programs (Fig 4b, main text).


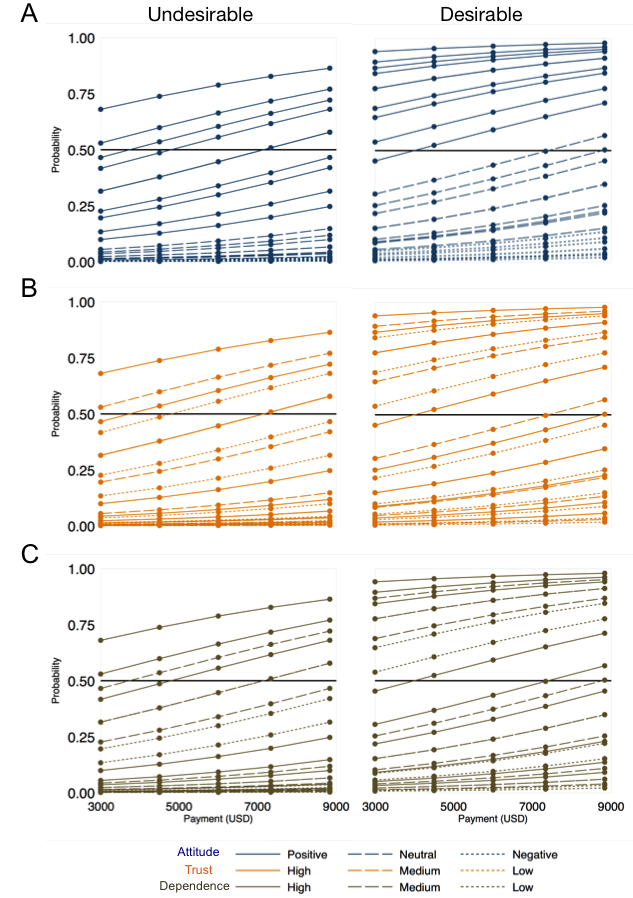


Figure S2. Comparing undesirable (left panel) to desirable (right panel) programs, a noticeable increase in the number of approved programs across cost levels occurs. Of 27 programs (each line represents a single program prototype), only 5 undesirable programs would be approved at a 50% majority level, while 9 desirable programs would be approved. Compared to an undesirable program, a greater diversity of fishers are willing to accept a desirable program at a lower cost. (*A*) Achieving approval requires fishers with positive attitudes about the outcomes. (*B*) Desirable programs can draw support from medium and low trusters as well as (*C*) Fishers with differing degrees of livelihood dependence.
